# Surgical management of hepatocellular carcinoma with tumor thrombi in the inferior vena cava or right atrium

**DOI:** 10.1186/1477-7819-11-259

**Published:** 2013-10-05

**Authors:** Kenji Wakayama, Toshiya Kamiyama, Hideki Yokoo, Tatsuhiko Kakisaka, Hirofumi Kamachi, Yosuke Tsuruga, Kazuaki Nakanishi, Tsuyoshi Shimamura, Satoru Todo, Akinobu Taketomi

**Affiliations:** 1 Department of Gastroenterological Surgery 1, Hokkaido University Graduate School of Medicine, N-15, W-7, Kita-ku, Sapporo, Japan

**Keywords:** Hepatocellular carcinoma, Inferior vena cava, Right atrium, Tumor thrombus, Surgery

## Abstract

**Background:**

The prognosis for advanced hepatocellular carcinoma (HCC) with tumor thrombi in the inferior vena cava (IVC) or right atrium (RA) is poor, and there is no established effective treatment for this condition. Thus study aimed to evaluate the efficacy of surgical resection and prognosis after surgery for such cases.

**Methods:**

Between January 1990 and December 2012, 891 patients underwent hepatectomy for HCC at our institution. Of these, 13 patients (1.5%) diagnosed with advanced HCC with tumor thrombi in the IVC or RA underwent hepatectomy and thrombectomy. Data detailing the surgical outcome were evaluated and recurrence-free and overall survival rates were calculated using the Kaplan-Meier method.

**Results:**

Seven patients had an IVC thrombus and six had an RA thrombus. Extra-hepatic metastasis was diagnosed in 8 of 13 patients. Surgical procedures included three extended right lobectomies, three extended left lobectomies, five right lobectomies, and two sectionectomies. Right adrenal gland metastases were excised simultaneously in two patients. All IVC thrombi were removed under hepatic vascular exclusion and all RA thrombi were removed under cardiopulmonary bypass (CPB). Four patients (30.8%) experienced controllable postoperative complications, and there was no surgical mortality. The mean postoperative hospital stay for patients with IVC and RA thrombi was 23.6 ± 12.5 days and 21.2 ± 4.6 days, respectively. Curative resection was performed in 5 of 13 cases. The 1- and 3-year overall survival rates were 50.4%, and 21.0%, respectively, and the median survival duration was 15.3 months. The 1- and 3-year overall survival rates for patients who underwent curative surgical resection were 80.0% and 30.0%, respectively, with a median survival duration of 30.8 months. All patients who underwent curative resection developed postoperative recurrences, with a median recurrence-free survival duration of 3.8 months. The 1-year survival rate for patients who underwent noncurative surgery and had residual tumors was 29.2%, with a median survival duration of 10.5 months.

**Conclusions:**

Aggressive surgical resection for HCC with tumor thrombi in the IVC or RA can be performed safely and may improve the prognoses of these patients. However, early recurrence and treatment for recurrent or metastatic tumors remain unresolved issues.

## Background

Hepatocellular carcinoma (HCC) is a highly malignant tumor with a propensity for invading intrahepatic blood vessels such as the portal vein (PV) or hepatic vein in advanced stages [1]. Further extension of tumor thrombi from any of the three main hepatic veins or the right inferior hepatic vein can give rise to thrombi in the inferior vena cava (IVC) or right atrium (RA) [1-3]. Commonly, the prognosis of HCC patients presenting with IVC or RA thrombosis is extremely poor [4-6], and there is no established management for such cases [4,5,7-17]. Surgical removal of IVC and RA thrombi combined with hepatectomy is the only radical treatment to decrease the risk of systemic metastasis and sudden death due to pulmonary embolism or occlusion of the tricuspid valve with a tumor thrombus [18-20]. However, aggressive surgical resection is not common because the surgical approach to IVC and RA thrombi is considered complicated and hazardous and is applicable only in limited cases with good hepatic reserve [4,6,9,16,17]. Therefore, the efficacy of surgical treatment for HCC with IVC or RA thrombi remains unclear. In this study, we retrospectively investigated the surgical outcomes and prognoses of patients who underwent surgery for HCC with IVC or RA tumor thrombi in a single institution to clarify the safety and efficacy of surgical resection.

## Methods

### Patients and diagnoses

**Figure 1:**
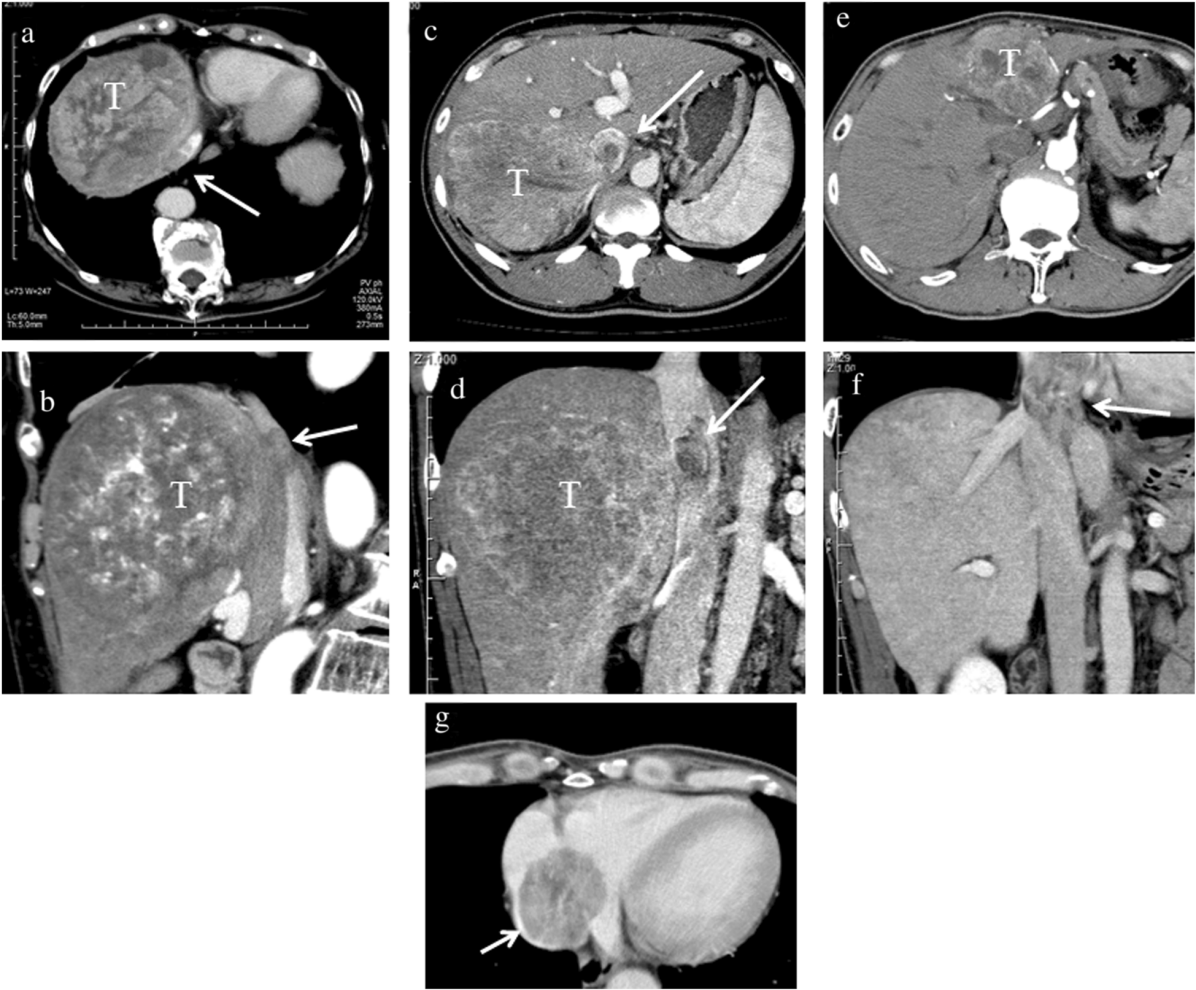
**Representative computed tomography findings of hepatocellular carcinoma (HCC) with inferior vena cava (IVC) and right atrium (RA) thrombi. **(**a** and **b**) A large HCC lesion in the right lobe (T), with a tumor thrombus arising from the right hepatic vein into the IVC (arrow). (**c** and **d**) HCC in the right lobe (T), with a tumor thrombus arising from the inferior right hepatic vein into the IVC (arrow). (**e**, **f**, and **g**) HCC in the left lobe (T), with a tumor thrombus arising from the left hepatic vein into the RA (arrow).

Between January 1990 and December 2012, 891 patients underwent hepatectomy for HCC at the Department of Gastroenterological Surgery, Hokkaido University, Japan. The diagnosis of HCC was determined by enhanced computed tomography (CT) and magnetic resonance imaging (MRI). IVC or RA thrombi were evaluated by CT (Figure 1). Among those studied, 13 patients (1.5%) diagnosed with advanced HCC and tumor thrombi in the IVC or RA underwent hepatectomy. This study was approved by the Institutional Review Board of the Hokkaido University School of Advanced Medicine.

The mean age at diagnosis was 63.4 years. The most common cause of HCC was hepatitis B virus (HBV) infection (53.8%), followed by hepatitis C virus (HCV) infection (15.4%). A total of 12 (92.3%) patients were male, and according to the Child-Pugh classification, all cases had Child-Pugh class-A disease. Six (46.2%) patients had a single tumor and seven (53.8%) had multiple tumors. The mean main tumor size was 11.8 cm, with nine tumors (69.2%) located in the right lobe and four (30.8%) in the left lobe. Extra-hepatic metastases were detected in eight of thirteen (61.5%) patients (five with lung, two with right adrenal gland and one with mediastinal lymph node metastases). Seven (53.8%) patients had an IVC thrombus and five (46.2%) had an RA thrombus (Table 1).

**Table 1:** Characteristics of patients and tumors

**Characteristic**	**Value**
Total number of patients	13
Age, years	
Mean ± SD (range)	63.4 ± 11.8 (37 to 86)
Sex	
Male/female	12/1
Hepatitis B virus	
Positive/negative	7/6
Hepatitis C virus	
Positive/negative	2/11
Child-Pugh classification	
A/B/C	13/0/0
Main tumor location	
Anterior/posterior/median/lateral section	5/4/3/1
Tumor size, cm	
Mean ± SD (range)	11.8 ± 4.3 (3.5–19)
Number of tumors	
Single/multiple	6/7
Extension of thrombus	
Inferior vena cava/right atrium	7/6
Preoperative extrahepatic metastases	
None/lung/adrenal gland/lymph nodes	5/5/2/1
Status of metastases after surgery	
Resected/regressed or stationary/progressed	3/1/4
Postoperative metastatic recurrence	
Liver/lung/lymph node/adrenal gland/inferior vena cava/brain	8/7/4/2/2/2

The tumor thrombus arose from the right hepatic vein in four patients (30.8%), middle hepatic vein in three (23.1%), left hepatic vein in one (7.7%), inferior right hepatic vein in two (15.4%), right hepatic vein combined with the middle hepatic vein in one (7.7%), and right hepatic vein combined with the inferior right hepatic vein in one (7.7%). In one patient with right adrenal gland metastasis, the tumor thrombus arose from the right adrenal vein (7.7%). Two patients had a mural thrombus and eleven had a massive thrombus. The massive thrombi in 10 patients did not completely occlude the IVC because circinate or arc-like luminal flow in the IVC around the tumor thrombi was present and the outflow canals of the intact hepatic veins were maintained. The tumor thrombus of one patient completely occluded the IVC inferior to the influx of hepatic veins accompanied by an aggregating blood thrombus. The outflow canals of the intact hepatic veins were severely narrowed but not completely occluded. One patient with a thrombus completely occluding the IVC and two patients with a massive IVC thrombus suffered preoperative renal insufficiency, and two had evident leg edema (Table 2).

**Table 2:** Characteristics of tumor thrombi

**Patients age/sex**	**Involved veins**	**Extent of thrombus**	**Advance of thrombus**	**Symptoms associated with the thrombus**
68/M	RHV	Massive	RA	Renal insufficiency/lower limb edema
57/M	RAdV	Massive	IVC	Renal insufficiency
70/M	RHV	IVC occlusive	RA	Renal insufficiency/lower limb edema
86/F	RHV	Massive	IVC	(−)
68/M	IRHV	Mural	IVC	(−)
66/M	RHV	Massive	RA	(−)
37/M	IRHV	Massive	IVC	(−)
56/M	RHV/IRHV	Massive	IVC	(−)
51/M	LHV	Massive	RA	(−)
72/M	MHV	Mural	IVC	(−)
59/M	LHV/MHV	Massive	RA	(−)
69/M	MHV	Massive	IVC	(−)
65/M	MHV	Massive	RA	(−)

*M* male, *RHV* Right hepatic vein, *RAdV* right adrenal vein, *IRHV* inferior right hepatic vein, *LHV* left hepatic vein, *MHV*, middle hepatic vein, *IVC* inferior vena cava, *RA* right atrium. (−), no symptom.

### Surgical procedures

A lobectomy was performed for patients with an indocyanine green retention rate at 15 minutes after injection (ICG R_15_) of <15% and total bilirubin levels of <1.5 mg/dl without ascites. Patients with an ICG R_15_ of 15 to 20% and total bilirubin levels of 1.5 to 2.0 mg/dl were eligible for sectionectomy according to our criteria [21]. The type of surgical procedure was selected based on Couinaud’s classification [22] and included three extended right hepatectomies, three extended left hepatectomies, five right hepatectomies, and two sectionectomies accompanied by thrombectomies. The right adrenal gland was resected simultaneously in two patients with right adrenal gland metastases (Table 3).

**Table 3:** Surgical procedure

	**Inferior vena**	**Right atrium**
	**cava thrombus**	**thrombus**
	**(n = 7)**	**(n = 6)**
Surgical procedure		
Extended right hepatectomy	1	1
Extended right hepatectomy + right adrenectomy	0	1
Right hepatectomy	3	1
Right hepatectomy + right adrenectomy	1	0
Extended left hepatectomy	1	2
Sectionectomy	1	1
Inflow vascular control		
Hepatic vascular exclusion	7	0
Cardiopulmonary bypass	0	4
CPB + portal vein/inferior vena cava to superior vena cava bypass	0	2
Vascular wall reconstruction		
Simple closure	7	4
Patch reconstruction	0	2

Hepatic resections were performed with an ultrasonic dissector using the Pringle maneuver in all cases. All IVC thrombi were removed under hepatic vascular exclusion (HVE). Before thrombectomy, hepatic transection was performed and the IVC was clamped below and above the liver. The IVC thrombus was excised en-bloc from the incised IVC, with satisfactory visualization of the intraluminal space under HVE. The IVC incision was closed by a simple continuous suture without a patch. All RA thrombi were removed under cardiopulmonary bypass (CPB). Following a laparotomy, a median sternotomy was performed to prepare for prompt CPB in anticipation of an undesirable pulmonary tumor embolism from a dislodged thrombus. Hepatic transection was then performed. The liver was handled gently, particularly if the thrombus had a long, thin neck, to prevent dissemination of tumor thrombi. Then, the superior vena cava (SVC) and IVC below the liver were clamped and blood flow was bypassed to the ascending aorta via an oxygenator. The RA was incised and the thrombus was excised en-bloc under direct vision. In most cases, the RA was reconstructed by simple sutures, but in two cases, an invaded RA wall was partially excised and reconstructed using an artificial graft or pericardial patch. In addition to CPB, one patient with complete IVC occlusion accompanied by severe obstruction of intact hepatic outflow and one patient with tumor thrombi that arose from two major hepatic veins showed gross hepatic congestion due to outflow block at surgery. These cases needed extracorporeal bypass from the portal vein (PV) and IVC to SVC (Table 3). In all cases, thrombi were intraoperatively monitored by transesophageal echocardiography. In this study, we defined curative resection as macroscopic complete excision of the tumors, including metastatic lesions.

### Follow up

The median duration of follow up was 11.2 (range, 1.8 to 51.8) months. Hospital death was defined as death occurring within 30 days of the first hospitalization. After surgery, CT or MRI was performed at 1- to 3-month intervals to determine recurrence. Data on surgical outcomes, postoperative management, recurrence, treatment of recurrence, and survival was analyzed for all cases.

### Statistical analysis

Survival rates were analyzed by the Kaplan-Meier method and statistical significance was determined by the log-rank test using JMP Pro 10.0.0 software (SAS, Cary, NC, USA). Significance was defined as *P* <0.05.

## Results

### Surgical outcomes and postoperative complications

With regard to patients with an IVC thrombus, the mean surgical duration was 349 ± 30 minutes, the median blood loss was 950 ± 100 ml, and the mean HVE duration was 8.8 ± 3.1 minutes. Two of seven (28.6%) patients needed blood transfusions. No patient required an ICU stay, and the mean postoperative hospital stay was 23.6 ± 12.5 days. After surgery, one patient experienced biloma and one experienced controllable ascites. With regard to patients with an RA thrombus, the mean surgical duration was 608 ± 169 minutes, the median blood loss was 6540 ± 5404 ml, and the mean CPB duration was 32.2 ± 18.3 minutes. Five of six (83.3%) patients needed blood transfusions. The mean postoperative ICU stay was 1.7 ± 0.8 days and the mean postoperative hospital stay was 21.2 ± 4.6 days. After surgery, one patient experienced acute renal failure and one experienced atrial fibrillation, but these patients recovered with medical therapy. There was no postoperative mortality. All IVC and RA thrombi were excised completely. Curative resection was performed in five of thirteen (38.5%) cases (Table 4).

**Table 4:** Surgical outcomes

	**Inferior vena cava thrombus**	**Right atrium thrombus**
**Surgical duration (minutes)**		
Mean ± SD (range)	349 ± 30 (288 to 377)	608 ± 169 (449 to 911)
Blood loss (ml)		
Median ± SE (range)	950 ± 100 (750 to 1,520)	6540 ± 5404 (1,050 to 35,820)
Blood transfusion		
Yes/no	2/5	5/1
HVE time (minutes)		
Mean ± SD (range)	8.8 ± 3.1 (8 to 13)	-
CPB time (minutes)		
Mean ± SD (range)	-	32.2 ± 18.3 (4 to 54)
Curative resection		
Yes/no	3/4	2/4
ICU stay (days)		
Mean ± SD (range)	-	1.7 ± 0.8 (0–2)
Hospital stay (days)		
Mean ± SD (range)	23.6 ± 12.5 (14 to 48)	21.2 ± 4.6 (16 to 28)
Complications		
Yes/no	2 (ascites, 1; biloma, 1)/5	2 (ARF, 1; Af, 1)/4

*HVE* hepatic vascular exclusion, *CPB* cardiopulmonary bypass, *ARF* acute renal failure, *Af* atrial fibrillation.

### Postoperative management

Among the five patients (38.5%) who underwent curative resection, adjuvant systemic chemotherapy was administered to four. The chemotherapeutic agents used in combination included intravenous 5-fluorouracil (5-FU; 500 mg weekly) and peroral tegafur uracil (UFT; 300 mg daily) in three patients and peroral UFT (300 mg daily) in one. One patient was followed up without adjuvant chemotherapy.

Tumors remained after surgery in eight (61.5%) patients, including lung metastases in four, intrahepatic metastases in two, both intrahepatic and lung metastases in one, and mediastinal lymph node metastases in one. Residual lung metastases were treated with oral administration of UFT in two patients, 5-FU + UFT in one patient, and oral administration of tegafur gimeracil oteracil potassium (S-1) followed by surgical resection in one patient. Unresectable intrahepatic metastases were treated with UFT in two patients and transarterial chemoembolization (TACE) in one patient. A patient with residual mediastinal lymph node metastasis received radiation after surgery.

### Recurrence and survival

All five patients who underwent curative resection experienced postoperative recurrences. Intrahepatic recurrences appeared in all five patients, lung metastases in four, intra-IVC metastases in one, and left adrenal gland metastases in one patient. The median recurrence-free survival duration of the patients who underwent curative resection was 3.8 months. Intrahepatic recurrences were treated with TACE in three patients, radiofrequency ablation (RFA) in two, and radiotherapy in one patient. Lung metastases were treated with systemic chemotherapy in three patients (5-FU + UFT in two, cisplatin (CDDP) + S-1 followed by oral administration of sorafenib in one patient), and surgical resection in one patient. Left adrenal gland metastases were surgically excised.

Among the eight patients who underwent noncurative resection, four of five with lung metastases exhibited progression of the metastases. In one patient, lung metastasis was resected but recurred after resection. Intrahepatic residual tumors in three patients progressed after surgery; however, mediastinal lymph node metastases treated by irradiation remained unchanged. Among these eight patients, seven experienced further dissemination of the tumor to new locations, including the lung in three, lymph nodes in three, brain in two, IVC in one, and adrenal gland in 1 (Tables 1 and 5).

**Table 5:** Characteristics and prognosis of patients

**Patients age/sex**	**Tumor thrombus**	**Residual tumor**		**Metastatic recurrence**		**Outcome (cause of death)**
		**Hepatic**	**Distant**	**Hepatic**	**Distant**	
68/M	RA	(−)	(−)	(+)	(+) (lung, Ad)	30.8 months; dead (cancer)
57/M	IVC	(−)	(−)	(+)	(−)	10.1 months; dead (cancer)
70/M	RA	(+)	(−)		(+) (lung)	9.1 months; dead (cancer)
86/F	IVC	(−)	(−)	(+)	(+) (lung, IVC)	15.3 months; dead (cancer)
68/M	IVC	(−)	(−)	(+)	(+) (lung, LN)	51.8 months; alive
66/M	RA	(−)	(+) (lung)	(+)	(+) (LN)	11.2 months; dead (cancer)
37/M	IVC	(−)	(+) (lung)	(−)	(+) (Ad, LN, Brain)	10.5 months; dead (cancer)
56/M	IVC	(−)	(+) (lung)	(−)	(−)	29.3 months; alive
51/M	RA	(−)	(+) (lung)	(+)	(+) (Brain)	8.5 months; dead (cancer)
72/M	IVC	(+)	(+) (lung)		(+) (LN)	1.9 months; dead (cancer)
59/M	RA	(−)	(−)	(+)	(+) (lung)	16.5 months; alive
69/M	IVC	(−)	(+) (LN)	(+)	(+) (lung, IVC)	16.0 months; alive
65/M	RA	(+)	(−)		(+) (lung)	7.6 months; alive

*Ad* adrenal gland, *IVC* inferior vena cava, *LN* lymph node.

**Figure 2:**
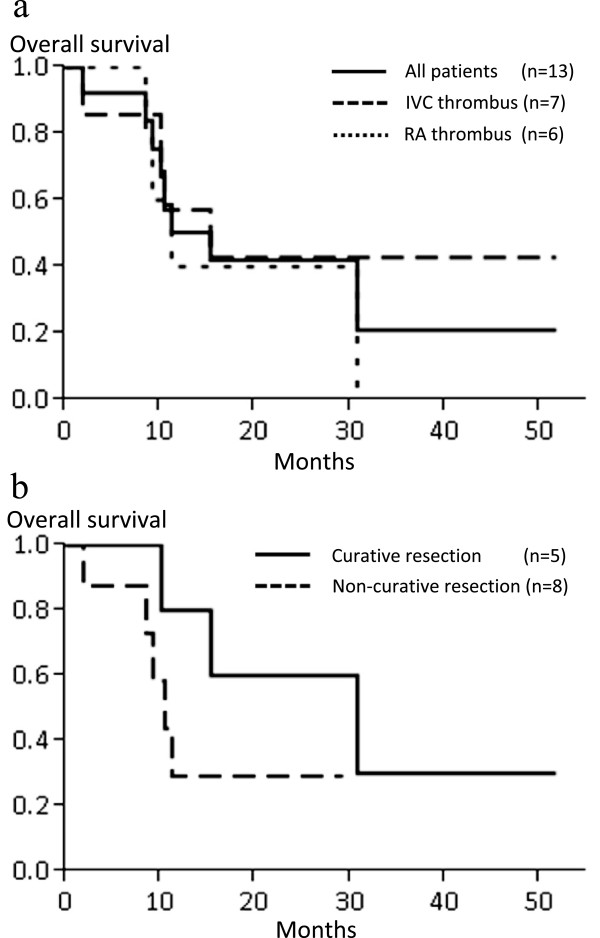
**Survival curve for patients with hepatocellular carcinoma (HCC) and inferior vena cava (IVC) and right atrium (RA) thrombi. (a)** Overall survival curve for all patients (n = 13), patients with IVC thrombus, and patients with RA thrombus. *P* = 0.6056 by log-rank test, IVC thrombus versus RA thrombus. **(b)** Overall survival curve for patients who underwent curative surgical resection and noncurative resection. *P* = 0.2103 by log-rank test, curative resection versus noncurative resection.

The 1-, and 3-year overall survival rates for all 13 patients were 50.4% and 21.0%, respectively, and the overall median survival duration was 15.3 months. The cause of postoperative death in all patients was cancer, which remained at surgery or recurred after surgery (Table 5). The overall survival rate for patients with IVC thrombi was 57.1% at 1 year and 42.9% at 3 years, with median survival duration of 15.3 months. The 1-year overall survival rate for patients with RA thrombi was 40.0%, with median survival duration of 11.2 months. There was no significant difference between the IVC thrombi and RA thrombi groups (Figure 2a). The survival rates for patients who underwent curative surgical resection were 80.0% at 1 year and 30.0% at 3 years, with a median survival time of 30.8 months. Meanwhile, the 1-year survival rate for patients who underwent noncurative surgery and had residual tumors was 29.2%, with a median survival time of 10.5 months (Figure 2b). The longest survival time was 51.8 months for patients who underwent complete resection and 29.3 months (to date) for those who underwent incomplete resection, and they are still alive (Table 5).

## Discussion

IVC and RA tumor thrombi arising from HCC are uncommon and are found in approximately 3 to 4% of HCC patients [2,23]. It is recognized that tumor invasion into intrahepatic vessels, such as the portal or hepatic veins, is an important prognostic factor for patients with HCC [24]. In particular, the prognosis of patients presenting with ICV or RA thrombi is extremely dismal [6]. Although surgical treatments as well as nonsurgical treatments such as TACE, radiotherapy, and chemotherapy are reported, optimal therapeutic management of IVC and RA thrombi has not been established because of the paucity of data [5,7,8,10-16]. Some reports demonstrate the potential benefit of surgical resection, but there are few reports that consolidate the efficacy of a surgical approach because IVC and RA thrombi are rare and because these reports are typically case reports or descriptions of a small number of patients [4,9,16,17,20,25,26]. Reports detailing the surgical treatment of RA thrombi are particularly rare, and to our knowledge this is the first report on the surgical treatment of IVC and RA thrombi, including six cases of RA thrombectomy, from a single institute.

It is generally assumed that liver resection combined with IVC or RA thrombectomy is a challenging and hazardous procedure that involves a high surgical risk. According to past reports, hepatectomy together with IVC or RA thrombectomy was associated with a high morbidity of 40% and a high mortality of 15% [4,15]. However, recent surgical innovations such as the inflow vascular control method together with refinement of the assessment of preoperative hepatic reserve have improved the safety of hepatectomy and thrombectomy procedures [26,27]. This progress has encouraged us to accept the challenge of aggressive surgical treatment for IVC and RA thrombi.

Effective control of intraoperative hemorrhage plays a crucial role in hepatectomy procedures combined with IVC or RA thrombectomy, because the degree of bleeding is a major predictive factor for operative morbidity and mortality [21]. In this study, hepatic parenchymal transection was routinely performed prior to thrombectomy using the Pringle maneuver. IVC occlusion at the suprahepatic portion with bulky tumor thrombi evokes Budd-Chiari syndrome and massive hepatic congestion [28]. In this study, we observed hepatic congestion in patients with outflow obstruction of spared hepatic veins by a massive tumor thrombus. Furthermore, occlusion of two of three major hepatic veins by venous invasion induced hepatic congestion, even though the spared hepatic vein was not obstructed. We used extracorporeal bypass from the PV and IVC to SVC to decompress the liver parenchyma and decrease bleeding during hepatic transection in two patients with an RA thrombus [29]. We performed IVC thrombectomy under a favorable field with good bleeding control by HVE. The duration of HVE, which could trigger hemodynamic deterioration, was short enough. Although CPB was mandatory for RA thrombectomy and was accompanied with a larger amount of blood loss and a higher rate of blood transfusion, patients with RA thrombi required minimal ICU stays and shorter postoperative hospitalization. These procedures contributed to a low incidence of postoperative non-serious complications that were medically manageable. Furthermore, we did not observe any operative mortality in this study. Therefore, hepatectomy with IVC or RA thrombectomy, although technically challenging, can be performed safely with appropriate inflow vascular control for patients with good hepatic reserve. Because almost all thrombi had capsules and did not adhere to the wall of the IVC or RA, they were simply removed by thrombectomy without wall resection. Although some authors indicate the efficacy of IVC resection, the benefits are controversial [30]. We experienced two cases of intra-IVC recurrence after surgery (Table 5); therefore, the management of such tumor thrombi should be reconsidered.

The prognosis of HCC patients with IVC or RA tumor thrombi is extremely poor. Earlier observations revealed a median survival duration after diagnosis of 1 to 5 months for untreated patients [4,15,31]. Although there is no consensus on the therapeutic options for HCC with IVC or RA thrombi, nonsurgical treatments such as TACE, as well as radiotherapy and chemotherapy, have been attempted. Previous reports concerning the therapeutic benefits of TACE with or without radiotherapy revealed insufficient results, with a median survival duration of 9.2 months (range, 4.2 to 18.4 months) [4,8,11-13,32]. Currently, the outcome of systemic chemotherapy for HCC has been disappointing, although sorafenib, which is the only effective agent against HCC, demonstrated a slightly better prognosis of 10.7 months in patients with unresectable HCC [33]. Some case reports have suggested the efficacy of surgical resection, and, recently, Wang *et al*. reported the significant superiority of a surgical approach to HCC with IVC or RA thrombi, with a median survival duration of 19 months, compared with TACE with or without chemoradiotherapy or no treatment [4,6,9,16,17,20,26,27],[34]. This study included 56 patients, of whom 25 underwent surgery, although 7 had an RA thrombus and only 3 underwent surgical resection for the same [4]. Therefore, the therapeutic benefit of surgical resection for HCC with an RA thrombus remains unclear. In the present study, the median overall survival duration of patients with an IVC or RA thrombus was comparable at 15.3 months and 11.2 months, respectively (Figure 2a). This finding indicates the equivalent therapeutic efficacy of surgical resection for RA or IVC thrombi. These results for patient survival are slightly worse than those of the previous study [4] because our study included patients who underwent non-curative resection. The median survival duration of patients who underwent curative resection was 30.8 months, which is longer than that in the previous report [4] (Figure 2b). This result also surpasses nonsurgical treatment with sorafenib, which resulted in median survival duration of 8.1 months in patients with macrovascular invasion [35].

All patients who underwent curative surgical resection experienced local recurrence or distant metastasis in the early postoperative phase, despite the fact that almost all patients received adjuvant chemotherapy (Table 5). It has been recognized that the poor prognosis of HCC with tumor thrombi in the IVC or RA is strongly related to a high incidence of postoperative recurrence at a relatively early stage, even after curative surgery. In this study, most patients who underwent curative resection developed postoperative lung metastases and intrahepatic recurrence at an equal rate. Preoperative or intraoperative dissemination of tumor cells to the lung can contribute to postoperative metastatic recurrence. To prevent potential intraoperative dissemination by intraoperative handling, some authors indicated the benefit of separated thrombectomy before hepatic transection [36]. However, it could be technically difficult to remove a thrombus en-bloc without up-front hepatic transection; therefore, further improvements in surgical techniques are required. To date, there is no clear modality established for preventing HCC recurrence [4,16,37]. The efficacy of preoperative radiotherapy is indicated for PV tumor thrombi [38]; however, the benefit for IVC or RA thrombi is unclear and there remains a risk of thrombi dislodgment during radiation. In this study, locally recurrent tumors were controlled by TACE or RFA and distant metastatic tumors were treated by chemotherapy with or without radiotherapy or surgical excision if resectable. These vigorous repetitive treatments contribute to improvement in survival, even after recurrence.

Surgical resection is commonly contraindicated for patients with unresectable metastatic tumors because incomplete resection is a crucial factor for poor prognosis [6]. However, hepatic lesions, but not distant metastasis, are the major factors influencing poor prognosis for death in the early postoperative phase [39]. On the basis of the fact that the survival duration of patients with IVC or RA thrombi is extremely short with nonsurgical treatment, distant metastasis itself should not be considered a contraindication for surgery. In this study, eight patients underwent noncurative surgery, and the median survival duration of 10.5 months was relatively better than that for patients who underwent nonsurgical treatment or no treatment in previous studies, including patients who received sorafenib, which is the only highly evidenced agent for advanced HCC treatment and results in median survival duration of 8.9 months in patients with extrahepatic metastases [4,8,11-13,15,30-32,35]. Recent reports indicate the efficacy of aggressive treatment for HCC metastases, including those to the lung, adrenal gland, and lymph nodes, by surgery or radiotherapy [40-42]. In this study, a patient with lung metastases at primary surgery underwent resection of the metastases and survived for 29.3 months. These findings indicate that reductive surgical resection can be justified in patients with IVC or RA thrombi accompanied by distant metastases or unresectable intrahepatic metastases. Control of the life-threatening progression of intrahepatic HCC and prevention of unexpected death by pulmonary embolism would give these patients a chance to undergo multidisciplinary treatments for improving survival. Therefore, if intrahepatic HCC and IVC or RA thrombi can be totally or partially resected, surgical resection may be beneficial.

The limitations of this study include its retrospective design, its single-center design, the small sample size, and patient heterogeneity. Because IVC and RA thrombi associated with HCC are rare, a multicenter prospective study with a large patients sample is necessary to definitively establish the benefits of surgical management.

## Conclusions

In conclusion, surgical resection of HCC with IVC or RA thrombosis can be performed safely with appropriate inflow vascular control in patients with good hepatic reserve. We suggest that aggressive surgical resection may be more beneficial than existing therapeutic modalities; however, early recurrence and treatment of recurrent or metastatic tumors remain unresolved issues. Further studies on adjuvant therapies and establishment of therapeutic strategies for recurrent and metastatic tumors are important challenges to improve survival.

## Abbreviations

Af: Atrial fibrillation; ARF: Acute renal failure; HBV: Hepatitis B virus; CDDP: Cisplatin; CPB: Cardiopulmonary bypass; CT: Computed tomography; 5-FU: 5-fluorouracil; HCC: Hepatocellular carcinoma; HCV: Hepatitis C virus; HVE: Hepatic vascular exclusion; ICG R15: Indocyanine green retention rate at 15 minutes after injection; IRHV: Inferior right hepatic vein; IVC: Inferior vena cava; LHV: Left hepatic vein; MHV: Middle hepatic vein; MRI: Magnetic resonance imaging; PV: Portal vein; RA: Right atrium; RAdV: Right adrenal vein; RFA: Radio frequency ablation; RHV: Right hepatic vein; S-1: Tegafur gimeracil oteracil potassium; SVC: Superior vena cava; TACE: Transarterial chemoembolization; UFT: Tegafur uracil.

## Competing interests

The authors have no conflicts of interest to declare.

## Authors’ contributions

KW and TK designed the research, KW, TK, HY, TK, HK, YT, KN, TS, ST, and AT contributed to acquisition of data, and KW and TK analyzed and interpreted data. All authors read and approved the final manuscript.

## References

[B1] Okuda K Hepatocellular carcinoma: clinicopathological aspects J Gastroenterol Hepatol 1997 12 S314 S318 10.1111/j.1440-1746.1997.tb00515.x 9407352

[B2] Kojiro M Nakahara H Sugihara S Murakami T Nakashima T Kawasaki H Hepatocellular carcinoma with intra-atrial tumor growth. A clinicopathologic study of 18 autopsy cases Arch Pathol Lab Med 1984 108 989 992 6095786

[B3] Tse HF Lau CP Lau YK Lai CL Transesophageal echocardiography in the detection of inferior vena cava and cardiac metastasis in hepatocellular carcinoma Clin Cardiol 1996 19 211 213 10.1002/clc.4960190314 8674258

[B4] Wang Y Yuan L Ge RL Sun Y Wei G Survival benefit of surgical treatment for hepatocellular carcinoma with inferior vena cava/right atrium tumor thrombus: results of a retrospective cohort study Ann Surg Oncol 2013 20 914 922 10.1245/s10434-012-2646-2 22956071

[B5] Chun YH Ahn SH Park JY Kim-do Y Han KH Chon CY Byun SJ Kim SU Clinical characteristics and treatment outcomes of hepatocellular carcinoma with inferior vena cava/heart invasion Anticancer Res 2011 31 4641 4646 22199343

[B6] Le Treut YP Hardwigsen J Ananian P Saisse J Gregoire E Richa H Campan P Resection of hepatocellular carcinoma with tumor thrombus in the major vasculature. A European case–control series J Gastrointest Surg 2006 10 855 862 10.1016/j.gassur.2005.12.011 16769542

[B7] Sun JH Zhang YL Nie CH Chen LM He JD Wang WL Zheng SS Long-term survival after chemoembolization of metastatic right atrial tumor thrombus as a presenting feature of hepatocellular carcinoma: a case study Oncol Lett 2012 3 975 977 22783375 10.3892/ol.2012.618 PMC3389639

[B8] Hou JZ Zeng ZC Zhang JY Fan J Zhou J Zeng MS Influence of tumor thrombus location on the outcome of external-beam radiation therapy in advanced hepatocellular carcinoma with macrovascular invasion Int J Radiat Oncol Biol Phys 2012 84 362 368 10.1016/j.ijrobp.2011.12.024 22381903

[B9] Jung H Lee KU Shin WY Ahn H Treatment outcomes of surgical resection for hepatocellular carcinoma with inferior vena cava invasion and/or thrombosis Hepatogastroenterology 2011 58 1694 1699 21940334 10.5754/hge10653

[B10] Matsuda M Shiba S Asakawa M Kono H Fujii H Complete remission of multiple recurrent hepatocellular carcinomas by oral administration of enteric-coated tegafur/uracil in a patient with huge hepatocellular carcinoma extending to the inferior vena cava after hepatic resection: analysis of mRNA expression of fluoropyrimidine metabolism enzymes in the primary tumor Int J Clin Oncol 2009 14 245 248 10.1007/s10147-008-0820-0 19593617

[B11] Chern MC Chuang VP Cheng T Lin ZH Lin YM Transcatheter arterial chemoembolization for advanced hepatocellular carcinoma with inferior vena cava and right atrial tumors Cardiovasc Intervent Radiol 2008 31 735 744 10.1007/s00270-008-9342-4 18427894

[B12] Cheng HY Wang XY Zhao GL Chen D Imaging findings and transcatheter arterial chemoembolization of hepatic malignancy with right atrial embolus in 46 patients World J Gastroenterol 2008 14 3563 3568 10.3748/wjg.14.3563 18567087 PMC2716621

[B13] Sugiyama S Beppu T Ishiko T Takahashi M Masuda T Hirata T Imai K Hayashi H Takamori H Kanemitsu K Hirota M Murakami R Baba Y Oya N Yamashita Y Baba H Efficacy of radiotherapy for PV and IVC tumor thrombosis in unresectable HCC Hepatogastroenterology 2007 54 1779 1782 18019717

[B14] Zeng ZC Fan J Tang ZY Zhou J Qin LX Wang JH Sun HC Wang BL Zhang JY Jiang GL Wang YQ A comparison of treatment combinations with and without radiotherapy for hepatocellular carcinoma with portal vein and/or inferior vena cava tumor thrombus Int J Radiat Oncol Biol Phys 2005 61 432 443 10.1016/j.ijrobp.2004.05.025 15667964

[B15] Chang JY Ka WS Chao TY Liu TW Chuang TR Chen LT Hepatocellular carcinoma with intra-atrial tumor thrombi. A report of three cases responsive to thalidomide treatment and literature review Oncology 2004 67 320 326 10.1159/000081333 15557794

[B16] Fukuda S Okuda K Imamura M Imamura I Eriguchi N Aoyagi S Surgical resection combined with chemotherapy for advanced hepatocellular carcinoma with tumor thrombus: report of 19 cases Surgery 2002 131 300 310 10.1067/msy.2002.120668 11894035

[B17] Tanaka A Morimoto T Ozaki N Ikai I Yamamoto Y Tsunekawa S Kitai T Yamaoka Y Extension of surgical indication for advanced hepatocellular carcinoma: is it possible to prolong life span or improve quality of life? Hepatogastroenterology 1996 43 1172 1181 8908547

[B18] Papp E Keszthelyi Z Kalmar NK Papp L Weninger C Tornoczky T Kalman E Toth K Habon T Pulmonary embolization as primary manifestation of hepatocellular carcinoma with intracardiac penetration: a case report World J Gastroenterol 2005 11 2357 2359 15818754 10.3748/wjg.v11.i15.2357 PMC4305827

[B19] Rajasekharan C Ganga V A rare cause for acute cor pulmonale Case Rep Gastroenterol 2011 5 330 335 10.1159/000329348 21712949 PMC3124326

[B20] Sung AD Cheng S Moslehi J Scully EP Prior JM Loscalzo J Hepatocellular carcinoma with intracavitary cardiac involvement: a case report and review of the literature Am J Cardiol 2008 102 643 645 10.1016/j.amjcard.2008.04.042 18721529

[B21] Kamiyama T Nakanishi K Yokoo H Kamachi H Tahara M Yamashita K Taniguchi M Shimamura T Matsushita M Todo S Perioperative management of hepatic resection toward zero mortality and morbidity: analysis of 793 consecutive cases in a single institution J Am Coll Surg 2010 211 443 449 10.1016/j.jamcollsurg.2010.06.005 20822741

[B22] Strasberg SM Nomenclature of hepatic anatomy and resections: a review of the Brisbane 2000 system J Hepatobiliary Pancreat Surg 2005 12 351 355 10.1007/s00534-005-0999-7 16258801

[B23] Lee IJ Chung JW Kim HC Yin YH So YH Jeon UB Jae HJ Cho BH Park JH Extrahepatic collateral artery supply to the tumor thrombi of hepatocellular carcinoma invading inferior vena cava: the prevalence and determinant factors J Vasc Interv Radiol 2009 20 22 29 10.1016/j.jvir.2008.09.030 19026566

[B24] Llovet JM Bustamante J Castells A Vilana R Ayuso Mdel C Sala M Bru C Rodes J Bruix J Natural history of untreated nonsurgical hepatocellular carcinoma: rationale for the design and evaluation of therapeutic trials Hepatology 1999 29 62 67 10.1002/hep.510290145 9862851

[B25] Shivathirthan N Shimoda M Kosuge T Kato M Kijima H Sawada T Kubota K Recurrent hepatocellular carcinoma with tumor thrombus in right atrium - report of a successful liver resection with tumor thrombectomy using total hepatic vascular exclusion without concomitant cardiopulmonary bypass Hepatogastroenterology 2012 59 872 874 22469735 10.5754/hge10662

[B26] Nonami T Nakao A Harada A Kaneko T Kurokawa T Takagi H Hepatic resection for hepatocellular carcinoma with a tumor thrombus extending to inferior vena cava Hepatogastroenterology 1997 44 798 802 9222693

[B27] Togo S Shimada H Tanaka K Masui H Fujii S Endo I Sekido H Management of malignant tumor with intracaval extension by selective clamping of IVC Hepatogastroenterology 1996 43 1165 1171 8908546

[B28] Okuda K Kage M Shrestha SM Proposal of a new nomenclature for Budd-Chiari syndrome: hepatic vein thrombosis versus thrombosis of the inferior vena cava at its hepatic portion Hepatology 1998 28 1191 1198 10.1002/hep.510280505 9794901

[B29] Fortner JG Kallum BO Kim DK Surgical management of hepatic vein occlusion by tumor: Budd-Chiari syndrome Arch Surg 1977 112 727 728 10.1001/archsurg.1977.01370060059009 193461

[B30] Matsuda H Sadamori H Shinoura S Umeda Y Yoshida R Satoh D Utsumi M Onishi T Yagi T Aggressive combined resection of hepatic inferior vena cava, with replacement by a ringed expanded polytetrafluoroethylene graft, in living-donor liver transplantation for hepatocellular carcinoma beyond the Milan criteria J Hepatobiliary Pancreat Sci 2010 17 719 724 10.1007/s00534-010-0287-z 20425126

[B31] Florman S Weaver M Primeaux P Killackey M Sierra R Gomez S Haque S Regenstein F Balart L Aggressive resection of hepatocellular carcinoma with right atrial involvement Am Surg 2009 75 1104 1108 19927515

[B32] Zeng ZC Fan J Tang ZY Zhou J Wang JH Wang BL Guo W Prognostic factors for patients with hepatocellular carcinoma with macroscopic portal vein or inferior vena cava tumor thrombi receiving external-beam radiation therapy Cancer Sci 2008 99 2510 2517 10.1111/j.1349-7006.2008.00981.x 19032365 PMC11158789

[B33] Llovet JM Ricci S Mazzaferro V Hilgard P Gane E Blanc JF de Oliveira AC Santoro A Raoul JL Forner A Schwartz M Porta C Zeuzem S Bolondi L Greten TF Galle PR Seitz JF Borbath I Haussinger D Giannaris T Shan M Moscovici M Voliotis D Bruix J Sorafenib in advanced hepatocellular carcinoma N Engl J Med 2008 359 378 390 10.1056/NEJMoa0708857 18650514

[B34] Yogita S Tashiro S Harada M Kitagawa T Kato I Hepatocellular carcinoma with extension into the right atrium: report of a successful liver resection by hepatic vascular exclusion using cardiopulmonary bypass J Med Invest 2000 47 155 160 11019497

[B35] Bruix J Raoul JL Sherman M Mazzaferro V Bolondi L Craxi A Galle PR Santoro A Beaugrand M Sangiovanni A Porta C Gerken G Marrero JA Nadel A Shan M Moscovici M Voliotis D Llovet JM Efficacy and safety of sorafenib in patients with advanced hepatocellular carcinoma: subanalyses of a phase III trial J Hepatol 2012 57 821 829 10.1016/j.jhep.2012.06.014 22727733 PMC12261288

[B36] Shirabe K Shimada M Tsujita E Maehara S Yamashita Y Rikimaru T Tanaka S Sugimachi K Thrombectomy before hepatic resection for hepatocellular carcinoma with a tumor thrombus extending to the inferior vena cava Int Surg 2001 86 141 143 11996069

[B37] Okada S How to manage hepatic vein tumour thrombus in hepatocellular carcinoma J Gastroenterol Hepatol 2000 15 346 348 10.1046/j.1440-1746.2000.02151.x 10824876

[B38] Kamiyama T Nakanishi K Yokoo H Tahara M Nakagawa T Kamachi H Taguchi H Shirato H Matsushita M Todo S Efficacy of preoperative radiotherapy to portal vein tumor thrombus in the main trunk or first branch in patients with hepatocellular carcinoma Int J Clin Oncol 2007 12 363 368 10.1007/s10147-007-0701-y 17929118

[B39] Kamiyama T Nakanishi K Yokoo H Kamachi H Tahara M Kakisaka T Tsuruga Y Todo S Taketomi A Analysis of the risk factors for early death due to disease recurrence or progression within 1 year after hepatectomy in patients with hepatocellular carcinoma World J Surg Oncol 2012 10 107 10.1186/1477-7819-10-107 22697061 PMC3407774

[B40] Hwang S Kim YH Kim DK Ahn CS Moon DB Kim KH Ha TY Song GW Jung DH Kim HR Park GC Namgoong JM Yoon SY Jung SW Park SI Lee SG Resection of pulmonary metastases from hepatocellular carcinoma following liver transplantation World J Surg 2012 36 1592 1602 10.1007/s00268-012-1533-0 22411088

[B41] Chua TC Morris DL Exploring the role of resection of extrahepatic metastases from hepatocellular carcinoma Surg Oncol 2011 21 95 101 21397495 10.1016/j.suronc.2011.01.005

[B42] Jiang W Zeng ZC Zhang JY Fan J Zeng MS Zhou J Palliative radiation therapy for pulmonary metastases from hepatocellular carcinoma Clin Exp Metastasis 2011 29 197 205 22173728 10.1007/s10585-011-9442-4

